# SOS genes are rapidly induced while translesion synthesis polymerase activity is temporally regulated

**DOI:** 10.3389/fmicb.2024.1373344

**Published:** 2024-03-26

**Authors:** Olaug Elisabeth Torheim Bergum, Amanda Holstad Singleton, Lisa Marie Røst, Antoine Bodein, Marie-Pier Scott-Boyer, Morten Beck Rye, Arnaud Droit, Per Bruheim, Marit Otterlei

**Affiliations:** ^1^Department of Clinical and Molecular Medicine, Norwegian University of Science and Technology (NTNU), Trondheim, Norway; ^2^Department of Biotechnology and Food Science, Norwegian University of Science and Technology (NTNU), Trondheim, Norway; ^3^Department of Molecular Medicine, CHU de Québec Research Center, Université Laval, Québec, QC, Canada; ^4^Clinic of Surgery, St. Olavs Hospital, Trondheim University Hospital, Trondheim, Norway; ^5^Clinic of Laboratory Medicine, St. Olavs Hospital, Trondheim University Hospital, Trondheim, Norway; ^6^BioCore - Bioinformatics Core Facility, Norwegian University of Science and Technology (NTNU), Trondheim, Norway

**Keywords:** SOS response, translesion synthesis, transcriptomics, proteomics, metabolomics, ciprofloxacin, *Escherichia coli*

## Abstract

The DNA damage inducible SOS response in bacteria serves to increase survival of the species at the cost of mutagenesis. The SOS response first initiates error-free repair followed by error-prone repair. Here, we have employed a multi-omics approach to elucidate the temporal coordination of the SOS response. *Escherichia coli* was grown in batch cultivation in bioreactors to ensure highly controlled conditions, and a low dose of the antibiotic ciprofloxacin was used to activate the SOS response while avoiding extensive cell death. Our results show that expression of genes involved in error-free and error-prone repair were both induced shortly after DNA damage, thus, challenging the established perception that the expression of error-prone repair genes is delayed. By combining transcriptomics and a sub-proteomics approach termed signalomics, we found that the temporal segregation of error-free and error-prone repair is primarily regulated after transcription, supporting the current literature. Furthermore, the heterology index (i.e., the binding affinity of LexA to the SOS box) was correlated to the maximum increase in gene expression and not to the time of induction of SOS genes. Finally, quantification of metabolites revealed increasing pyrimidine pools as a late feature of the SOS response. Our results elucidate how the SOS response is coordinated, showing a rapid transcriptional response and temporal regulation of mutagenesis on the protein and metabolite levels.

## 1 Introduction

Exposure to certain antibiotics inflicts lethal DNA damage in bacteria. The fluoroquinolone ciprofloxacin traps gyrase on DNA and causes replication fork arrest and DNA breaks in *Escherichia coli* (Drlica et al., 2009). These breaks result in single-stranded DNA (ssDNA) that initiate the SOS response through RecA-ssDNA binding. The RecA-ssDNA nucleoprotein filament catalyzed the self-cleavage of LexA and subsequent transcription of the LexA-repressed SOS regulon, which includes more than 60 genes (Simmons et al., 2008). The heterology index (HI) describes the binding affinity of LexA to the SOS box, in which lower HI indicates higher similarity to a consensus LexA binding site and thus, higher affinity. In general, a gene must have an HI value below 15 to be considered a LexA-repressed gene (Lewis et al., 1994; Fernández de Henestrosa et al., 2000; Courcelle et al., 2001). Collectively, induction of the SOS response results in DNA repair, cell division arrest, and membrane potential disruption. DNA repair consists of two phases. The first phase is error-free and involves nucleotide excision repair and homologous recombination. The second phase follows if the DNA damage persists and is mainly performed by error-prone translesion synthesis (TLS) polymerases. In other words, the proteins in the SOS response are active at different times depending on the degree of DNA damage. The SOS response is crucial for cell survival, as mutants with reduced or no SOS response show higher sensitivity to antibiotics and other stressors (Miyabe et al., 2000; Sharma et al., 2013; Mo et al., 2016; Recacha et al., 2017).

Ever since the rise of high throughput omics technologies in the mid-1990s, omics research on bacterial DNA damage has mainly focused on transcriptomics using microarray (Courcelle et al., 2001; Gmuender et al., 2001; Shaw et al., 2003; Kaldalu et al., 2004; Cirz et al., 2006, 2007). Because RNA sequencing has improved sensitivity and dynamic range compared to microarray, and only a few studies have looked at proteomics or metabolomics (Belenky et al., 2015; Zampieri et al., 2017; Li et al., 2018), we wanted to explore the SOS response by combining these three omics, which, to our knowledge, has not been previously done. Integrating two or more omics provides a more global understanding of how genotype affects phenotype compared to single omics. Also, multi-omics has the potential to uncover how a single event influences downstream pathways and how the response is regulated. Several of the previous omics studies have used high antibiotic concentrations to achieve substantial cell death (Kaldalu et al., 2004; Cirz et al., 2006, 2007; Belenky et al., 2015). Since the main purpose of the SOS response is to increase species survival by promoting mutagenesis, we wanted to examine this response at a clinically and ecologically relevant antibiotic concentration, as this is more relevant for the development of antibiotic resistance.

In this study, we uncovered new insights into the coordinated response to ciprofloxacin-induced DNA damage in *E. coli* by using a highly controlled, longitudinal multi-omics study. Importantly, the experiments were performed in bioreactors to obtain controlled experimental conditions that gave highly reproducible results from three independent biological replicates (BR). Longitudinal sampling allowed us to follow the different stages of SOS activation over time. While transcriptomics allowed us to observe gene expression in response to ciprofloxacin, simultaneous analysis of a sub-proteomic fraction enriched in signaling proteins, herein called the signalome, highlighted changes in the activated protein pool as DNA damage accumulated. Unlike looking at the whole proteome, signalomics can capture changes in protein activation and deactivation that occur much faster than translation by binding to exposed ATP/GTP-binding motifs. Signalomics was performed by selective enrichment for activated proteins and protein complexes using the recently established multiplexed kinase inhibitor bead (MIB) assay for prokaryotes (Singleton et al., 2023). Finally, metabolomics revealed how DNA damage caused by ciprofloxacin changes the metabolite pools and suggests which pathways have increased or decreased flux. This multi-omics study showed that temporal segregation of different SOS activities is regulated after transcription which challenges previous publications that suggest temporal segregation of SOS gene expression (Janion, 2008; Simmons et al., 2008; Lima-Noronha et al., 2022). Additionally, we observed a negative correlation between HI and log_2_ fold change (LFC) of gene expression, and not the time of induction as outlined elsewhere (Goodman et al., 2016; Lima-Noronha et al., 2022).

## 2 Materials and methods

### 2.1 Bacterial strain and media

Experiments were performed with *E. coli* K-12 MG1655 in cation-adjusted Mueller-Hinton broth II (CAMHB; Becton Dickinson, United States). All pre-cultures of *E. coli* were inoculated from glycerol stock in CAMHB and grown overnight at 37°C and 250 rpm.

### 2.2 Minimal inhibitory concentration (MIC) assay

The MIC determination of ciprofloxacin (Sigma-Aldrich, United States) was performed according to standard susceptibility testing established by the Clinical and Laboratory Standards Institute (Cockerill et al., 2012), with adjustments as described earlier (Nedal et al., 2020). MICs were performed in triplicates and microtiter plates were inspected for visible growth after 24 h at 37°C.

### 2.3 Rifampicin resistance (Rif^R^) assay

Mutation frequency using the Rif^R^ assay was determined as previously described (Thi et al., 2011). Briefly, the *E. coli* pre-culture was diluted to OD_600_ = 0.0125 in CAMHB in a shake flask, followed by incubation at 37°C and 250 rpm to OD_600_ = 0.2. The culture was split into two shake flasks: one was treated with ciprofloxacin (12 ng/mL) and the other was left as an untreated control. The cultures were incubated for 120 min at 37°C and 250 rpm. Sampling was performed by withdrawing 2 mL bacterial suspension from each of the two cultures. Samples were washed twice by centrifugation (3,220 rcf, 10 min) and resuspended in fresh CAMHB. Resuspended samples were incubated overnight at 37°C and 250 rpm to resolve filaments formed by ciprofloxacin. Microscopy was used to confirm filament resolution (<5% filamentation). To determine CFU/mL, appropriate dilutions were plated on LB agar plates and incubated at 37°C for 24 h. Aliquots were also plated on LB agar plates containing 100 μg/mL rifampicin and incubated at 37°C for 48 h. Mutation frequency was calculated by dividing the number of rifampicin-resistant colonies per 10^8^ viable cells per mL (Rif^R^/10^8^ CFU/mL).

### 2.4 Batch setup

The batch setup was prepared using 1 L bioreactors (Applikon, Netherlands) with 1 L CAMHB. The pH probe was calibrated with pre-mixed solutions of pH 4 and pH 7. pH 7 was maintained by automatic titration of 3 M HCl and 4 M NaOH. The dissolved oxygen (DO) probe was calibrated to 100% DO in the bioreactor after the CAMHB reached 37°C with an aeration rate of 500 mL min^−1^ and 200 rpm stirring. The DO probe was flushed with nitrogen gas before use to ensure electrode sensitivity. Stirring was adjusted between 200 and 600 rpm to maintain a DO above 40%, thus, avoiding bacterial stress caused by oxygen depletion. The inflow air was sterile filtered using a 0.2 μm filter. O_2_ consumption and CO_2_ production were constantly monitored by analyzing the off-gas with the Prima Bench Top Process Mass Spectrometer gas analyzer (Thermo Fisher Scientific, United States). Excess foam formation was prevented by manually adding a silicone polymer antifoam (Sigma-Aldrich).

### 2.5 Ciprofloxacin treatment

The *E. coli* pre-culture was diluted to OD_600_ = 0.0125 in two separate bioreactors. This dilution ensured that the *E. coli* population from the pre-culture entered the log phase before ciprofloxacin treatment. One batch culture was treated with 12 ng/mL ciprofloxacin at OD_600_ = 0.2, while the remaining batch culture was left as the untreated control.

### 2.6 Sampling

Sampling from the bioreactor was conducted 1 min before treatment (approximately OD_600_ = 0.19) and 1, 10, 25, 50, 75, and 120 min after treatment. After the first timepoints, the 25 min intervals were selected to ensure a doubling of the population between the sampling. Samples were taken for live-dead staining, transcriptomics, signalomics and metabolomics. The samples, except for live/dead staining, were stored at −80°C until further processing.

Sampling was performed from 3 BRs, with one technical replicate (TR) per timepoint for transcriptomics and signalomics, and 4 TRs per timepoint for metabolomics.

#### 2.6.1 Live/dead staining

Samples were taken 120 min after OD_600_ = 0.2 and appropriately diluted. Diluted samples were washed three times with 0.85% NaCl, stained with Live/Dead BacLight bacterial viability kit (Invitrogen, United States), immobilized on agarose pads (0.1% w/v low-melt agarose), and inspected by microscopy (Zeiss Axio Imager Z.2, Germany). Counting of bacteria from images was performed in the software ImageJ v 1.51 (Schneider et al., 2012).

#### 2.6.2 Metabolomics

The sampling procedure was adapted from a previously described protocol optimized for *E. coli* (Thorfinnsdottir et al., 2023). At each sampling timepoint, OD_600_ was measured to determine the sampling volume. Eight OD units (1 OD unit corresponds to 1 mL bacterial culture with an OD_600_ = 1) were sampled per TR. Samples were filtered using Durapore^®^ polyvinylidene fluoride 0.45 μm filters (Millipore, United States) exposed to a vacuum pressure of 250 mbar below the ambient pressure. Filtered biomass was washed with Milli-Q H_2_O (10 mL, 37°C). Two filters were used per TR to reduce filtration times for the large sample volumes. Biomass from both filters were pooled into 15 mL ice-cold quenching solution (20:30:50 MeOH:ACN:H_2_O), frozen in N_2_ (*l*), and kept at −80°C until further processing.

Additionally, an aliquot of *E. coli* culture from several timepoints was filtered through a pre-weighted filter and washed with Milli-Q H_2_O (10 mL, 37°C), as described above. Filters were placed in a heating cabinet (110°C) and the weight was determined the following day. The cell density (OD_600_) was plotted against the cell dry weight (CDW) per L sampled cell culture. The CDW for each sample taken during the sampling period was calculated by interpolation.

#### 2.6.3 Signalomics

Based on the OD_600_ at each sampling timepoint, a volume of *E. coli* culture between 2 and 10 mL was centrifuged (4,500 rcf, 10 min, 4°C). The supernatant was discarded, and the pellet was frozen in N_2_ (*l*).

#### 2.6.4 Transcriptomics

Transcriptomics was sampled according to the protocol provided with RNeasy Mini kit (Qiagen, Germany). The sample volume was based on the OD_600_ at each sampling timepoint to ensure approximately 3.35 × 10^8^ cells per sample. The sample was immediately added to 2 volumes of RNA protect and vortexed. After minimum 5 min, the samples were centrifuged (4,500 rcf, 10 min). The supernatant was discarded and the pellet was frozen in N_2_ (*l*).

### 2.7 Sample processing

#### 2.7.1 Metabolomics

Sample processing was adapted from a previously described protocol (Thorfinnsdottir et al., 2023). Briefly, intracellular metabolites were extracted by cycling samples between −20°C EtOH and N_2_ (*l*) in three consecutive freeze–thaw cycles, with vortexing every 10 min during the thawing phase. Filters were removed and the cell debris was pelleted (4,500 rcf, 10 min, −9°C). The supernatants were transferred to a new tube, snap frozen in N_2_ (*l*), and lyophilized. Lyophilized extracts were reconstituted in 500 μL cold Milli-Q H_2_O and cleared by spin-filtration with a 10 kDa molecular cutoff (20,817 rcf, 10 min, 0°C). A mix of 80 μL centrifuged sample and 20 μL ^13^C-labeled ISTD extract from yeast was sent to analysis.

#### 2.7.2 Signalomics

Cell extracts were prepared by a combination of lysozyme treatment (1 mg/mL) and freeze–thaw cycling between N_2_ (*l*) and H_2_O (37°C). The MIB assay with on-column trypsinization was used to isolate ATP/GTP-binding proteins from 100 μL cell extract containing 1 mg/mL protein (Petrovic et al., 2017). The kinase inhibitor mix was previously optimized to pull down a larger portion of the bacterial signalome, and consisted of an equal mix of Purvalanol B (Tocris, United Kingdom), L-1 and L-3 (in-house) (Singleton et al., 2023). Samples were stored at −20°C until analysis.

#### 2.7.3 Transcriptomics

RNA was isolated using RNeasy Mini Kit (Qiagen), according to manufacturer’s protocol 4 (Enzymatic lysis and Proteinase K digestion of bacteria) and protocol 7 (Purification of total RNA) with on-column DNase (Qiagen) digestion. The RNA concentration in each sample was measured using NanoDrop-1000 spectrophotometer (Thermo Fisher Scientific). Samples were stored at −80°C until analysis.

### 2.8 Sample analysis

#### 2.8.1 Metabolomics

Capillary ion chromatography tandem mass spectrometry (capIC-MS/MS) was used to quantify phosphorylated metabolites, organic acids, and intermediates of the TCA cycle. Metabolite extracts were analyzed with a Xevo TQ-XS triple quadrupole mass spectrometer (Waters, United States), as previously described (Kvitvang and Bruheim, 2015), with modifications (Stafsnes et al., 2018).

#### 2.8.2 Signalomics

Signalomics sample analysis was performed by the Proteomics Core Facility at NTNU using liquid chromatography (LC) MS/MS. A timsTOF Pro 2 (Bruker Daltonics, United States) connected to a nanoElute (Bruker Daltonics) HPLC system was used to perform the LC–MS/MS analysis. Peptide separation was conducted using a Bruker PepSep column (25 cm x 75 μm x 1.5 μm) kept at 50°C. The LC used running buffers A (0.1% formic acid) and B (0.1% formic acid in acetonitrile) with a gradient ranging from 2% B to 40% B over 40 min at a flow rate of 250 nL/min. Subsequently, within 1 min, the gradient transitioned to 95% B and a flow rate of 300 nL/min, which was maintained for 9 min. The MS instrument was operated in data dependent acquisition parallel accumulation serial fragmentation (DDA-PASEF) mode with 10 PASEF scans per acquisition cycle, and accumulation and ramp times of 100 milliseconds each. The ‘target value’ was set to 20,000 and dynamic exclusion was activated and set to 0.4 min. The quadrupole isolation width was set to 2 Th for m/z < 700 and 3 Th for m/z > 800.

#### 2.8.3 Transcriptomics

Sequencing was conducted by the Genomics Core Facility at NTNU. RNA sequencing libraries were prepared using the QIAseq FastSelect 5S/16S/23S kit (Qiagen) for rRNA removal and the QIAseq stranded RNA Lib kit (Qiagen) for library construction, according to the manufacturer’s instructions.

Briefly, 500 ng total RNA was used as starting material. Removal of ribosomal RNA (rRNA) was conducted by a combined heat fragmentation (89°C for 7 min) and FastSelect hybridization protocol (75–4°C ramping process) where the FastSelect reagent inhibited reverse transcription of bacterial rRNA. Next, purification was conducted using QIAseq Beads followed by a first-strand synthesis using a RNase H- Reverse Transcriptase (RT) in combination with random primers, a second-strand synthesis, end-repair, A-addition, and adapter ligation. The second-strand synthesis was performed using 5’phosphorylated random primers which enable subsequent strand-specific ligation. DNA fragments were further enriched by CleanStart library amplification (15 cycles of PCR reaction). Finally, the libraries were purified using the QIAseq Beads, quantitated by qPCR using Collibri Library Quantification Kit (Thermo Fisher Scientific), and validated using Perkin Elmer DNA 1 K/12 K/Hi Sensitivity Assay LabChip on a Labchip GX instrument (Perkin Elmer, United States). The size range of the DNA fragments were measured to be in the range of 270 to 570 bp and peaked around 355 bp.

Prior to sequencing, libraries were normalized and pooled to 2.3 pM and subjected to clustering on three NextSeq 500 HO flow cells (Illumina, United States). Finally, single read sequencing was performed for 75 cycles on a NextSeq 500 instrument (Illumina), according to the manufacturer’s instructions. Base calling was done on the NextSeq 500 instrument by RTA v 2.4.6. FASTQ files were generated using bcl2fastq2 Conversion Software v 2.20.0.422 (Illumina). The sequencing depth was approximately 180×.

### 2.9 Data analysis

#### 2.9.1 Metabolomics

Data processing and absolute quantification was performed as earlier described (Røst et al., 2020) using the TargetLynx application manager of MassLynx v 4.1 (Waters) to interpolate calibration curves made with appropriate dilutions of analytical grade standards (Sigma-Aldrich). The response factor of the corresponding U^13^C-isotopologues were used to correct the standard and sample extract response factors. Extract concentrations were normalized to the CDW, which was calculated from interpolation of the OD_600_ vs. CDW (g/L) curve. The data is available on Zenodo at DOI: 10.5281/zenodo.10277458.

Further statistical analysis in MetaboAnalyst v 5.0 (Xia et al., 2009) replaced missing values with 1/5 of the minimum value of the respective metabolite. An unpaired *T*-test with unequal variance determined differential enriched metabolites with a false discovery rate (FDR) < 0.05 which are presented as LFC compared to control.

#### 2.9.2 Signalomics

MS data were processed using MaxQuant v 2.1.3.0 for label-free quantification (LFQ) of proteins (Tyanova et al., 2016). The following search parameters were used: the digestion enzyme was specified as trypsin with a maximum of 2 missing cleavages, variable modifications were set to oxidation (M), acetylation of protein N-terminal, and deamination (NQ), and fixed modifications were set to carbamidomethyl (C). LFQ min. Ratio count was set to 1. Samples were queried against the imported *E. coli* K-12 reference proteome (including isoforms) downloaded from the UniProt website^1^ (accessed on April 06, 2022) and Andromeda, MaxQuant’s internal contaminants database. FDR for protein and peptide identification was set to 1%. Only unique peptides were used for definite protein group identification. The area under the peak curve was integrated to obtain peak abundances. The total abundance of all peptides identified for each protein during each run was used to normalize the abundance in every protein group using the LFQ algorithm (Cox et al., 2014) with minimum peptides ≥1.

The LFQ values were analyzed in R v 4.1.2 using the DEP package v 1.18.0 from Bioconductor (Zhang et al., 2018). The data was normalized using variance stabilizing transformation. Each timepoint was analyzed separately. Proteins identified in at least 2 BRs for either ciprofloxacin or control samples were included in the analysis. Missing values for proteins showing a similar trend of being upregulated (≥0) or downregulated (≤0) after ciprofloxacin treatment were imputed by random draws from a Gaussian distribution centered around a minimal value (“MinProb”) as MNAR (missing not at random), while the remaining missing values were imputed by k-nearest neighbor method (“knn”) as MAR (missing at random). DEP uses protein-wise linear models combined with empirical Bayes statistics to find differentially enriched proteins. Differentially enriched proteins were defined with FDR < 0.1 and presented as LFC compared to the control.

The raw data and results have been deposited in the ProteomeXchange Consortium via the PRIDE (Perez-Riverol et al., 2019) partner repository with the dataset identifier PXD047394.

#### 2.9.3 Transcriptomics

Processing of the sequence data was done with the ProkSeq v 2.0 (Mahmud et al., 2020) program using Docker, with the *E. coli* K-12 MG1655 reference genome ASM584v2 (RefSeq: GCF_000005845.2) and corresponding reference transcriptome. ProkSeq is a docker based, full RNA-Seq pipeline for prokaryotes, which includes quality assessment, alignment, gene counting, and analysis. The count tables countFile.csv, countFileNucleotideAvgCount.csv and countFile_TPM_CPM.tsv were exported from ProkSeq and used for further analysis.

Count files were analyzed in R using the quasi-likelihood *F*-test in EdgeR v 3.36.0 from Bioconductor (Robinson et al., 2010). The FDRs were calculated using the Benjamini-Hochberg procedure. Poorly expressed genes were filtered out, and library size was normalized using trimmed mean of *M*-value (TMM). Differentially expressed genes were defined with FDR < 0.05 and presented as LFC compared to the control.

The raw and processed data have been deposited in NCBI’s Gene Expression Omnibus (GEO) (Edgar et al., 2002) and are accessible through GEO Series accession number GSE249682.^2^

#### 2.9.4 Multi-omics integration

Analysis of the longitudinal multi-omics data was performed using timeOmics v 1.6.0 from Bioconductor (Bodein et al., 2022b). Significant variables compared to the control at timepoints up to 120 min were extracted for signalomics (FDR < 0.1), metabolomics (FDR < 0.05) and transcriptomics (1–50 min, FDR < 0.05; 75–120 min, FDR < 0.05, LFC < −1.5 or > 1.5). Linear mixed effect model splines (lmms; removed from the CRAN repository and archived on 2020-09-11) with basis “cubic” for transcriptomics and signalomics and basis “p-spline” for metabolomics was applied on these variables for imputation of missing timepoints. The data was clustered by multi-block partial least squares (MB-PLS), and the clustering with highest silhouette coefficient was chosen for further analysis. Gene ontology (GO) and Kyoto Encyclopedia of Genes and Genomes (KEGG) enrichment analysis (FDR < 0.05) with clusterProfiler v 4.2.0 from Bioconductor (Wu et al., 2021) was performed separately on each omics obtained from the MB-PLS clusters.

Exploration of multi-omics networks was performed with netOmics v 1.4.0 from Bioconductor (Bodein et al., 2022a). The SOS response network was constructed using interactions from BioGRID database v 4.4.209 (Oughtred et al., 2021), where interactions between genes have an experimental system type “genetic” and interactions between proteins “physical.” Next, the separate gene and protein networks were combined by connecting proteins to the corresponding genes. The nodes in the final networks were colored according to the maximum achieved LFC during the sampling period, either positive or negative, compared to the control. The nucleotide metabolism networks were constructed using interactions from graphite v 1.44.0 from Bioconductor (Sales et al., 2012). Metabolites and genes, and interactions between them, were obtained from KEGG pathways of purine metabolism (eco00230) and pyrimidine metabolism (eco00240). The protein network was added by connecting proteins to the corresponding genes. The nodes in the final networks were colored according to their LFC at 120 min. The networks were imported into Cytoscape (Shannon et al., 2003) for adjustments.

## 3 Results and discussion

### 3.1 Sub-MIC ciprofloxacin treatment led to substantial changes at multiple omics levels without affecting the growth rate

The SOS response is well studied across several bacterial species using different DNA-damaging agents (Simmons et al., 2008; Maslowska et al., 2019); however, most omics studies focus only on gene expression. Thus, we performed a longitudinal multi-omics study of ciprofloxacin-treated *E. coli* K-12 MG1655 using three omics; transcriptomics, proteomics, and metabolomics. A sub-MIC concentration (12 ng/mL, ¾ × MIC) allowed us to understand the DNA damaging effects of ciprofloxacin while avoiding substantial cell death. After 120 min, the ciprofloxacin-treated culture had 92% live cells compared to 96% in the untreated control. Despite high survival, initial experiments showed more than a doubling of the Rif^R^ mutation frequency with ¾ × MIC ciprofloxacin; thus, verifying that the SOS response was activated (Supplementary Figure S1).

**Figure 1:**
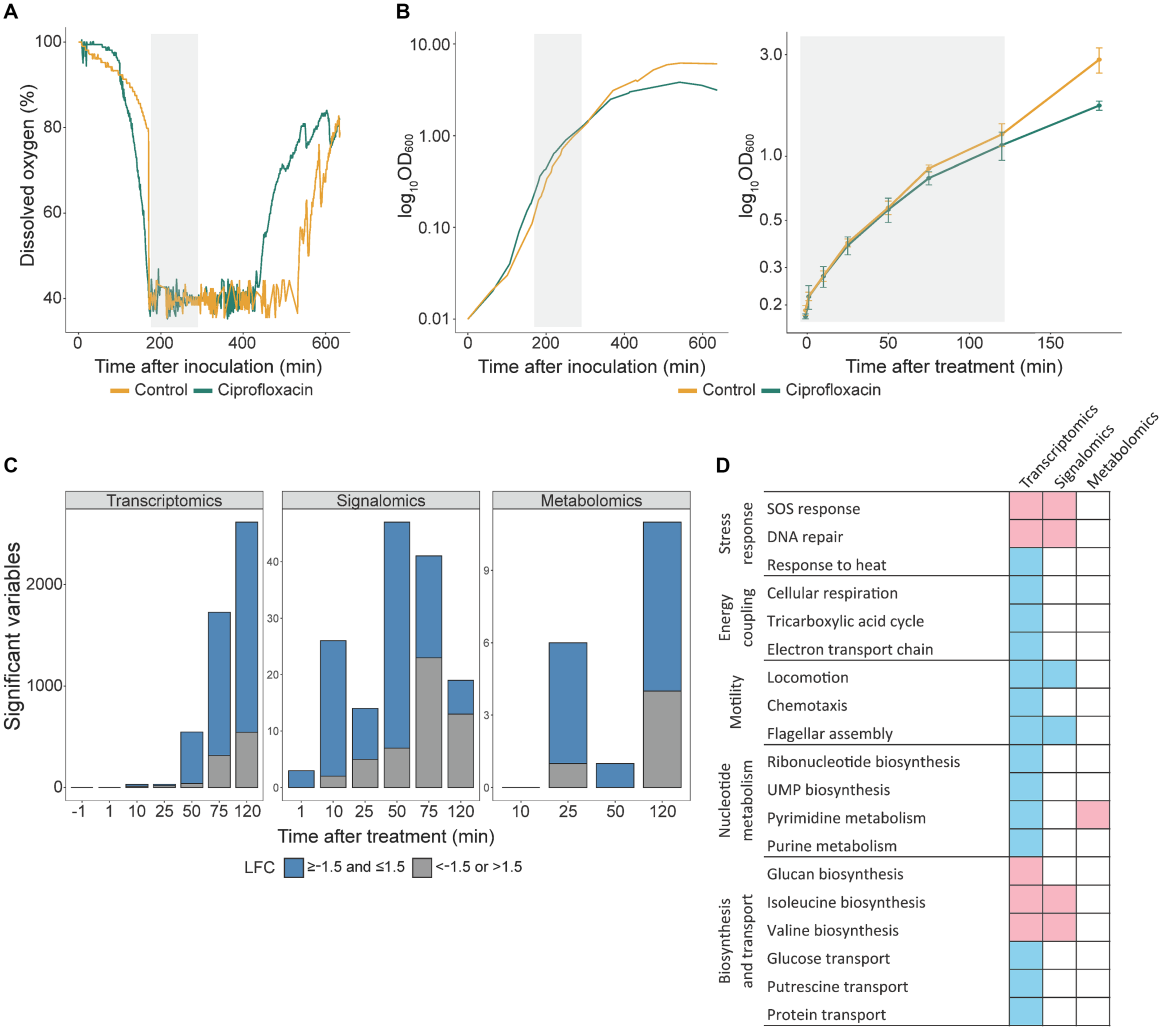
Ciprofloxacin reduces the maximum cell density and changes transcription, protein activation and metabolite pools in a time-dependent manner. **(A)** Dissolved oxygen and **(B)** semi-log growth curve of ciprofloxacin-treated *E. coli* K-12 MG1655 and untreated control. The grey area indicates the sampling period. The data in **(A)** and (**B**, left) are from biological replicate (BR) 2 but representative of all BRs. (**B**, right) The mean growth curve for all BRs during the sampling period. Mean ± SD, *n* = 3. **(C)** Number of significant variables for transcriptomics (FDR < 0.05), signalomics (FDR < 0.1) and metabolomics (FDR < 0.05), grouped with log_2_ fold change (LFC) of < −1.5 or >1.5, and ≥−1.5 and ≤1.5. **(D)** Enriched GO terms (FDR < 0.05) and KEGG pathways (FDR < 0.05) from significant changes in the transcriptome, signalome and metabolome. The significant variables were analyzed separately from each of the two multi-omics clusters and the resulting enriched GO terms and KEGG pathways are colored according to an increasing (pink) or decreasing (blue) trend over time.

Published omics studies on bacterial DNA damage are mostly performed in shake flasks or culture tubes where the growth conditions are difficult to control (Shaw et al., 2003; Kaldalu et al., 2004). In this study, we used bioreactors which enabled us to carefully control and monitor growth conditions such as dissolved oxygen (DO), temperature and pH. Increasing DO indicates reduced growth as the bacterial culture uses less oxygen. The DO in the ciprofloxacin-treated culture started to increase 100 min before the control, and the treated culture obtained a maximum OD_600_ of 4 compared to OD_600_ of 6 for the control (Figures 1A,B), i.e., the maximum achievable cell density was reduced following exposure to ciprofloxacin. Although post-treatment morphological changes such as filamentation might influence the OD, an unchanged OD_600_ to cell dry weight ratio after treatment compared to the untreated control (data not shown) suggests that the reduced maximum OD_600_ arises from inhibited growth.

Most studies focusing on transcriptomics after DNA damage included a maximum of three timepoints (Gmuender et al., 2001; Shaw et al., 2003; Kaldalu et al., 2004; Cirz et al., 2006, 2007); thus, the six timepoints included here allows for a more detailed understanding of how the SOS response progresses over time. We sampled frequently during the first 2 hours to closely capture SOS activation as well as include the same timepoints as previous studies (Courcelle et al., 2001; Gmuender et al., 2001; Shaw et al., 2003; Kaldalu et al., 2004; Cirz et al., 2006, 2007). Notably, all sampling timepoints were during the exponential growth phase as DO levels were still low and the cell density had not yet plateaued (Figures 1A,B). In addition, samples were taken from three independent BRs. The samples taken 1 min before treatment had no differentially expressed genes (Figure 1C, left panel), which illustrates similar growth conditions between the BRs and shows that the substantial alterations at all three omics levels are due to ciprofloxacin.

In this study, we chose a signalomics approach rather than a whole proteome approach because the total protein pool is not able to capture subtle changes in protein activation or deactivation that occur in response to stimuli such as DNA damage. Reversible activation of kinases depends on the conformation of the ATP-binding motif. The baits in the MIB assay are compounds that mimic kinase inhibitors by binding to the activated ATP-binding motif of the kinases (Duncan et al., 2012; Roskoski, 2016). The commercial kinase inhibitor Purvalanol B used in the MIB assay has been shown to also bind to proteins that are not kinases but possess an ATP/GTP-binding motif (Bantscheff et al., 2007; Petrovic et al., 2017). We also expect the two in-house compounds to bind to most proteins with an ATP/GTP binding motif and proteins in complex with these, based on their ability to pull down around 1,000 proteins each (Singleton et al., 2023). The extracted proteins thus include kinases as well as other ATP/GTP-binding proteins and represent an enrichment of signaling proteins. Increased protein pulldown reflects increased activation and/or expression, whereas decreased protein pulldown reflects degradation or deactivation. Based on the differential protein pulldown in the signalome profile, we can infer which processes are switched on or off after ciprofloxacin-induced DNA damage. From here on, the term activation will be used to indicate increased pulldown of a protein.

### 3.2 Ciprofloxacin activates the SOS response and subsequently impacts energy coupling, motility, and nucleotide metabolism

We clustered the longitudinal multi-omics data to study how ciprofloxacin affected gene expression, protein activation, and metabolite pools during the sampling period (Figure 1C). Significant changes relative to the control included 1,093 genes (1–50 min, FDR < 0.05; 75–120 min, FDR < 0.05 and LFC < −1.5 or >1.5), 168 activated proteins (FDR < 0.1), and 14 metabolites (FDR < 0.05) (Supplementary Table S1). The ciprofloxacin response at 75 and 120 min gave a total of 2,955 genes with an FDR below 0.05, which covers 68% of the detected transcriptome. To avoid including an extensive number of transcripts in the clustering and instead study the most significant changes, we selected an LFC threshold of 1.5 at 75 and 120 min. We did not use an LFC threshold of 1.5 at earlier timepoints, as this would exclude most transcripts. The variables inside each of the resulting two clusters showed either an increasing or decreasing LFC over time (Supplementary Figure S2). While most transcripts and metabolites increased in LFC over time, protein activation decreased. The MIB assay pulls down ATP/GTP interacting proteins and proteins in complex with these. Thus, this does not cover the complete proteome. Many of the detected proteins in this study are not directly involved in the SOS response (Supplementary Figure S3), which may explain their decreased activity during the sampling period. On the other hand, we observed mostly increased gene expression while previous studies have reported mostly downregulated expression (Gmuender et al., 2001; Kaldalu et al., 2004; Cirz et al., 2006) or equal amounts of upregulated and downregulated expression (Cirz et al., 2007). This discrepancy from our results is likely due to the use of quinolone concentrations above MIC in previous studies, which might shut down additional cellular processes as bacterial cell death increases.

From the increasing and decreasing clusters, the changes in transcriptome and signalome were examined for enriched GO terms and metabolome changes for enriched KEGG pathways (Figure 1D, pink = increasing, blue = decreasing). A detailed overview of enriched GO terms including FDR and gene/protein ratio is shown in Supplementary Figure S4. As ciprofloxacin induces DNA damage, an activated SOS response was detected already after 10 min in both the transcriptome and signalome (Figure 1D). This supports that the SOS response is the primary response of ciprofloxacin treatment. Following the initial DNA damage, secondary responses observed after 50 min included multiple processes such as energy coupling, motility, and nucleotide metabolism (Figure 1D). The latter was also altered at the metabolome level.

### 3.3 SOS gene expression and protein activation are not always correlated

**Figure 2:**
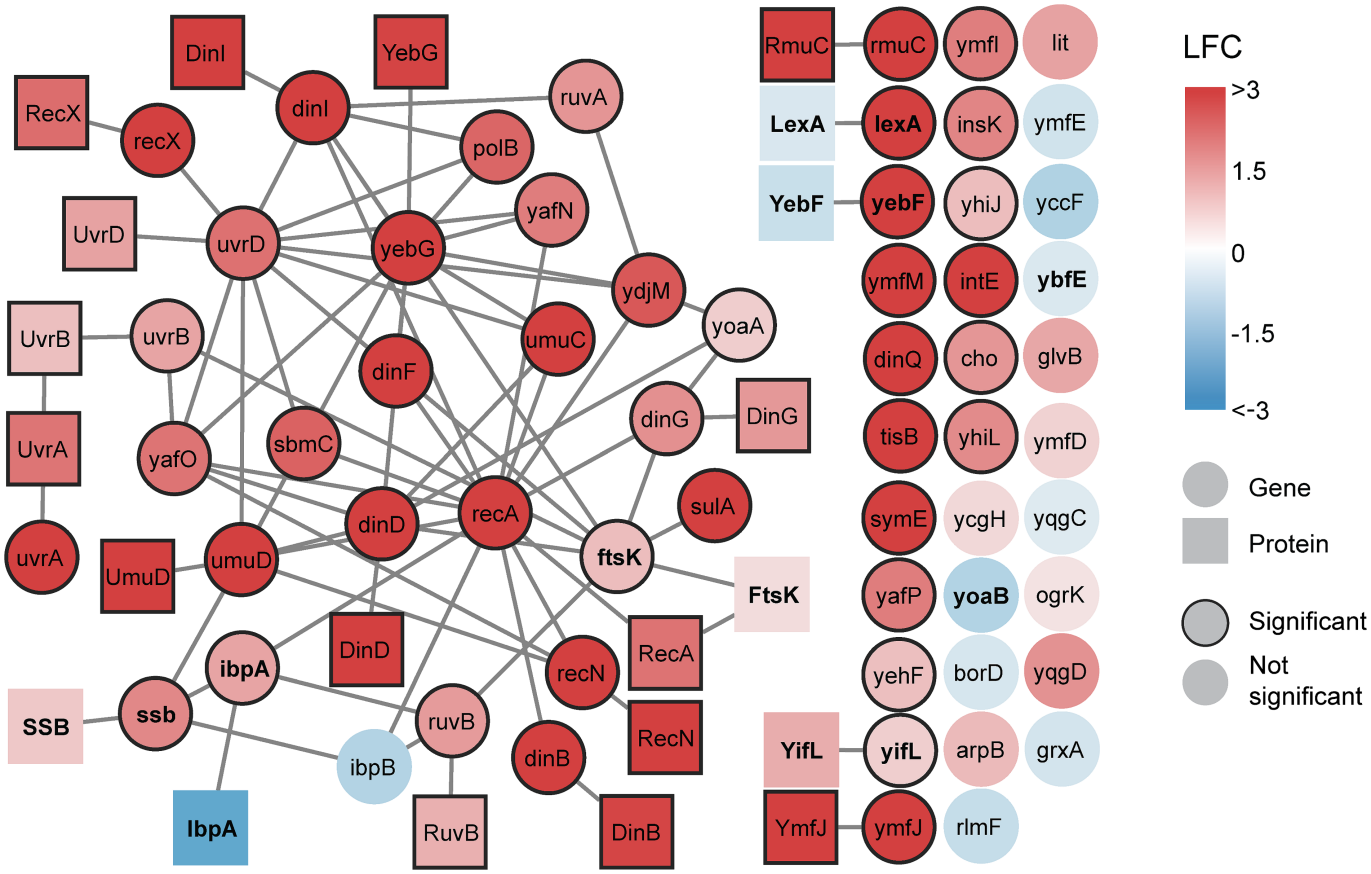
A multi-omics network of the SOS response reveals increasing gene expression and protein activation after DNA damage. All detected transcripts from the SOS gene list are included. Proteins are included if their corresponding gene is differentially expressed. Edges connecting genes represent genetic interactions and edges connecting proteins represent physical interactions. All interactions are sourced from BioGRID. Genes and proteins are colored based on maximum achieved significant log_2_ fold change (LFC), either positive or negative, during the time course (0–120 min). Significant nodes have borders. If no significant change is achieved (no border) during the time course, the nodes are colored based on maximum LFC during the time course. Genes and proteins discussed in the text are bold.

To further explore the SOS response, we analyzed the individual SOS genes by constructing a gene list with LexA-repressed genes based on a review by Simmons et al. (2008) and supplemented with additional genes (Stohl et al., 2003; Christensen-Dalsgaard et al., 2010; Thomassen et al., 2010; Sutera et al., 2021) (Supplementary Table S2). This gene list was used to select for SOS genes in the transcriptome and signalome data (Table 1). Next, the SOS genes and their corresponding protein products were visualized in a multi-omics network (Figure 2). This network clearly shows that most SOS genes and protein activation are significantly increased during the sampling period.

**Table 1:** Log_2_ fold change of gene expression and protein activation at sampled timepoints after ciprofloxacin treatment.

Gene/Protein	Omics	1min	10min	25min	50min	75min	120min
*arpB*	T	−0.38	−0.12	−0.16	−0.22	**1.00**	0.75
*borD*	T	0.06	−0.06	0.39	−0.13	−0.51	−0.48
*cho*	T	0.21	**0.92**	**0.93**	**1.14**	**1.59**	**1.49**
*dinB*	T	0.13	**1.69**	**1.82**	**2.74**	**2.92**	**3.13**
DinB	S					1.39	**2.92**
*dinD*	T	0.25	**1.66**	**2.61**	**4.22**	**4.19**	**4.47**
DinD	S			**2.88**	**3.66**	**5.77**	**5.34**
*dinF*	T	0.06	**1.33**	**1.78**	**3.04**	**1.83**	**2.98**
*dinG*	T	0.05	**0.98**	**1.17**	**1.63**	**1.45**	**1.69**
DinG	S		−0.39	−0.19	**1.56**	3.55	2.19
*dinI*	T	0.08	**1.91**	**3.08**	**3.62**	**3.70**	**3.24**
DinI	S	1.00	0.56	**1.59**	**2.63**	**2.72**	**3.15**
*dinQ*	T	0.29	0.34	0.84	**1.52**	**1.34**	**3.37**
*ftsK*	T	0.07	**0.58**	0.52	**0.94**	−0.10	0.07
FtsK	S	0.02	−0.11	0.20	0.41	0.45	0.34
*glvB*	T	0.24	0.92	0.17	0.96	**1.32**	**1.18**
*grxA*	T	−0.38	−0.02	0.16	−0.37	−0.10	−0.53
GrxA	S	0.40	−0.17	−0.01	−0.32	−0.56	−0.19
*ibpA*	T	0.01	0.33	1.55	**1.35**	0.12	−0.11
IbpA	S	0.16	−0.42	−2.21	−0.02	−0.04	0.53
*ibpB*	T	−0.18	0.24	1.22	0.80	0.76	−**1.01**
IbpB	S	0.26	−0.09	−0.32			0.17
*insK*	T	0.01	−0.09	0.12	−0.07	**1.41**	**1.87**
intE*	T	0.01	−0.32	−0.24	**0.70**	**3.56**	**4.31**
*lexA*	T	0.24	**1.53**	**1.65**	**3.38**	**3.46**	**3.48**
LexA	S	−0.05	−0.21	−0.44	0.09	−0.18	−0.06
lit*	T	0.14	−0.18	−0.13	−0.16	−0.45	**1.39**
*ogrK*	T	0.03	0.10	−0.09	0.17	0.34	−0.29
*polB*	T	0.02	**1.23**	**1.41**	**2.34**	**1.49**	**2.38**
*recA*	T	0.10	**1.77**	**2.22**	**3.35**	**3.76**	**3.78**
RecA	S	0.11	0.18	0.72	**1.76**	**1.28**	**2.16**
*recN*	T	0.31	**2.76**	**3.14**	**4.45**	**3.75**	**4.63**
RecN	S	−0.40	**2.47**	**2.91**	**4.59**	**6.65**	**6.79**
*recX*	T	0.14	**1.39**	**1.68**	**2.57**	**3.69**	**4.02**
RecX	S				**2.19**	2.21	2.26
*rlmF*	T	−0.01	−0.09	−0.02	−0.35	0.10	**−0.74**
RlmF	S	−0.06	0.36	0.02	−0.59	−0.09	−0.04
*rmuC*	T	0.17	1.05	**1.90**	**2.85**	**3.03**	**3.09**
RmuC	S	0.04	0.37	**2.03**	**3.65**	**4.08**	**3.69**
*ruvA*	T	0.13	**1.06**	**1.02**	**1.60**	**1.18**	**1.00**
*ruvB*	T	0.12	**0.95**	**0.80**	**1.50**	**1.06**	**0.65**
RuvB	S	0.06	0.12	0.30	**1.17**	0.19	1.02
*sbmC*	T	−0.04	1.21	**2.18**	**2.45**	**2.24**	**1.71**
*ssb*	T	−0.05	0.10	**0.80**	0.36	**1.82**	**1.37**
Ssb	S	−0.24	0.07	0.16	−0.05	0.77	0.41
*sulA*	T	0.19	**1.93**	**2.47**	**3.41**	**4.71**	**3.06**
*symE*	T	0.39	0.55	0.94	**1.75**	**2.92**	**3.22**
*tisB*	T	−0.01	0.84	**2.56**	**4.03**	**4.18**	**4.96**
*umuC*	T	0.02	**1.85**	**2.18**	**3.35**	**4.09**	**3.18**
*umuD*	T	0.07	**1.82**	**2.60**	**3.61**	**4.25**	**3.04**
UmuD	S					**3.00**	**3.87**
*uvrA*	T	0.04	**1.33**	**1.46**	**2.54**	**2.46**	**3.20**
UvrA	S	−0.11	0.08	**0.65**	**1.23**	**1.38**	**2.13**
*uvrB*	T	−0.01	**0.75**	**0.75**	**1.35**	**0.73**	**1.26**
UvrB	S	−0.22	0.17	0.32	**0.76**	0.92	0.81
*uvrD*	T	0.04	**0.84**	**1.47**	**1.62**	**2.07**	**2.19**
UvrD	S	−0.15	0.00	0.30	**1.39**	**1.09**	**1.25**
*yafN*	T	0.00	**0.95**	**0.98**	**1.75**	**1.58**	**2.00**
*yafO*	T	−0.16	0.79	1.04	**1.67**	**1.34**	**2.15**
*yafP*	T	−0.03	**0.95**	0.81	**1.88**	0.55	**2.00**
*ybfE*	T	0.09	0.05	−0.40	−0.43	−0.36	**−0.33**
YbfE	S	−0.46	0.53	−0.36	−0.41	1.88	0.69
*yccF*	T	0.05	0.31	0.28	−0.11	−0.34	**−1.05**
YccF	S		0.83		−0.05		
*ycgH*	T	−0.08	−0.16	−0.19	−0.46	0.37	0.50
*ydjM*	T	−0.04	**1.59**	**1.53**	**2.61**	**2.60**	**1.81**
*yebF*	T	0.15	**1.78**	**2.18**	**3.11**	**3.44**	**2.12**
YebF	S				−0.68	−0.28	−0.55
*yebG*	T	0.13	**1.81**	**2.53**	**3.11**	**4.06**	**2.93**
YebG	S	0.69			**2.93**	3.13	1.89
*yehF*	T	−0.12	0.15	0.93	**0.92**	**1.14**	0.80
*yhiJ*	T	0.05	0.43	−0.10	**0.94**	**0.71**	**1.01**
*yhiL*	T	0.34	0.10	0.06	0.80	**1.77**	**1.41**
*yifL*	T	−0.07	0.19	0.56	**0.67**	**0.94**	**1.15**
YifL	S	0.14	0.59	1.08	−0.78	1.22	
ymfD*	T	0.06	−0.07	0.26	0.42	0.08	0.61
ymfE*	T	−0.08	−0.20	0.08	0.36	−0.30	−0.57
ymfI*	T	−0.09	0.09	−0.52	−0.07	**1.00**	**1.98**
ymfJ*	T	−0.30	0.18	0.20	**1.34**	**4.05**	**4.83**
YmfJ	S						**3.81**
*ymfM*	T	0.04	0.11	0.14	1.44	**4.00**	**4.77**
*yoaA*	T	0.02	**0.52**	0.65	**0.68**	0.04	−0.25
*yoaB*	T	0.03	0.11	0.28	0.01	−0.43	**−1.01**
*yqgC*	T	−0.01	0.16	0.34	−0.16	−0.15	−0.37
*yqgD*	T	0.07	0.08	1.65	−0.38	0.36	−0.12
*yqgC*	T	−0.01	0.16	0.34	−0.16	−0.15	−0.37
*yqgD*	T	0.07	0.08	1.65	−0.38	0.36	−0.12

Includes SOS genes listed in Supplementary Table S2. Bold values have an FDR < 0.05 for genes and FDR < 0.1 for proteins. T, transcriptomics; S, signalomics. ^*^e14 prophage encoded genes (Mehta et al., 2004).

Ciprofloxacin treatment did not yield significant differential expression of 16 LexA-repressed genes (Figure 2, circles without border). Among these non-significant genes, only the uncharacterized genes *ybfE* and *yoaB* possess an SOS box likely to bind LexA (HI <15) (Fernández de Henestrosa et al., 2000; Courcelle et al., 2001). The remaining genes are classified as LexA-repressed based on increased expression after UV irradiation in wild-type *E. coli* but not in a mutant strain unable to express genes under LexA control (Courcelle et al., 2001). Interestingly, most of the non-significant genes do not interact with other genes in the multi-omics network (Figure 2). This may indicate that there is little experimental evidence of these genes’ involvement in the SOS response besides the UV irradiation study (Courcelle et al., 2001). These genes might therefore be specifically induced upon UV exposure or require more DNA damage than produced by a sub-MIC dose of ciprofloxacin.

Overall, the data from the signalome analysis correlated with the transcriptome data as expression of SOS genes resulted in a significant increase of activated protein product. However, increased expression did not always yield activated protein product. An example is *lexA*, where the RecA-ssDNA nucleoprotein filament mediates LexA self-cleavage, thus likely preventing an increase in levels of activated LexA (Figure 2). Also, despite increased expression of *ssb*, enrichment of the single-strand binding protein (SSB) was not significant. This is in agreement with another study where induced *ssb* expression did not alter SSB levels after treatment with a quinolone (Perrino et al., 1987). In addition, increased gene expression did not activate the cell division protein FtsK, heat-shock protein IbpA, and the functionally unknown proteins YifL and YebF (Figure 2). This suggests protein degradation, post-transcriptional regulation, or absent post-translational activation. These proteins might only become translated or activated in the SOS response if a certain degree of DNA damage is acquired or after the sampling period used in this study. Since protein activation occurs much faster than transcription and translation, the presence of inactive proteins allows for a swift response when their activity is needed.

### 3.4 The HI negatively correlates with maximum LFC

**Figure 3:**
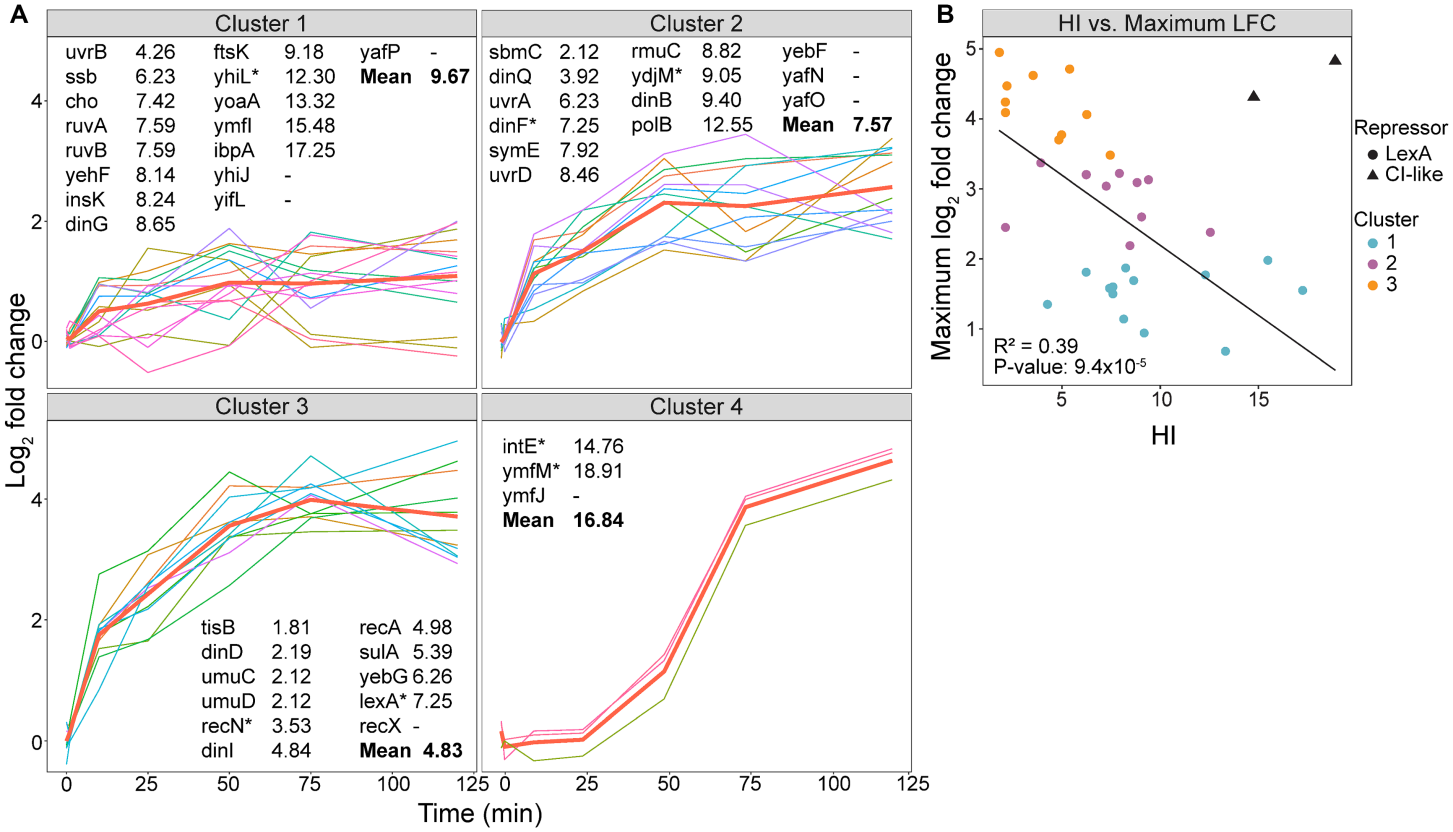
Expression of SOS genes varies in time of induction and maximum log_2_ fold change (LFC). **(A)** Hierarchical clustering of differentially expressed SOS genes (1–50 min, FDR < 0.05; 75–120 min, FDR < 0.05 and LFC < −1.5 or >1.5) into four clusters with an average silhouette width of 0.39 (cluster 1, 0.40; cluster 2, 0.22; cluster 3, 0.47; and cluster 4, 0.74), shown with the mean expression from each cluster (bold red line). Genes are listed with the heterology index (HI) of LexA-binding sequences (Courcelle et al., 2001). Gene^*^ has >1 LexA-binding sequences, where the lowest HI is listed. **(B)** Scatterplot of the maximum LFC as a function of the HI. A linear regression model was used to create the trend line. The e14 prophage encoded genes (triangles) were not included in the linear regression. These genes are under regulation of a CI-like repressor.

Our experimental setup allowed us to identify longitudinal patterns in the expression of the SOS genes, including time of induction, maximum LFC, and if the expression plateaus or starts to decrease over the time course. Based on hierarchical clustering, the differentially expressed SOS genes were grouped into four clusters (Figure 3A, bolded red lines represent the expression mean of each cluster). Genes in clusters 2 and 3 show a rapid induction at 10 min, whereas genes in cluster 4 are induced at 50 min. Cluster 1, however, displays a more heterogenous expression pattern. Most genes within cluster 1 are induced at 10 min, with expression levels either increasing or stabilizing throughout the sampling period. In contrast, genes encoding heat-shock protein IbpA, cell division protein FtsK, and DNA-helicase YoaA exhibit rapid induction at 10 min but with declining expression after 50 min. Additionally, genes encoding transposase InsK and the functionally unknown YhiJ show induced expression only after 50 min (Figure 3A and Table 1). This suggests that these genes may be subject to additional transcriptional regulation beyond LexA derepression in response to DNA damage.

A significant difference in HI values was observed between cluster 1 and 3 (*p =* 0.001) and cluster 2 and 3 (*p =* 0.03), but not between cluster 1 and 2 (*p =* 0.2). Genes with a high HI are not completely repressed in undamaged cells, and a basal level of the corresponding protein is expected to be active independent of the SOS response. For example, *ssb* has an HI of 6.23 and the presence of ~7,000 SSBs contributes to normal replication in undamaged cells (Simmons et al., 2008). In comparison, genes with a low HI are under strong LexA control and function mainly in the SOS response. Among these is the TLS polymerase operon *umuDC* with an HI of 2.12, which has very low expression in undamaged cells (Woodgate and Ennis, 1991). Based on this, the genes with a low HI, and thus, low basal levels under normal conditions, are expected to reach a higher LFC after SOS activation than those with a high HI. Accordingly, our data suggested that a decreasing mean HI for cluster 1 to 3 correlated with stronger induction of expression (Figure 3A). A scatterplot further supports this observation as it shows a significant correlation between HI and maximum LFC (Figure 3B).

Some of the LexA-repressed genes deviated from the expected correlation between HI and maximum LFC. This could be due to: (i) some genes possess multiple SOS boxes with different HI (Courcelle et al., 2001), and the interplay between these SOS boxes might affect the LexA repression differently than those with only one SOS box, (ii) the distance between the SOS box and the start codon of the gene affects the expression in undamaged cells (Mérida-Floriano et al., 2021), and this distance varies between LexA-repressed genes, and (iii) additional repressors could repress the gene’s transcription even after SOS activation. An example of the latter is *uvrB* which has a low HI (4.26), but still has a low maximum LFC (Figure 3A). As *uvrB* is repressed by both LexA and DnaA (Wurihan et al., 2018), the low maximum LFC indicates that DnaA might continue to repress some of its expression after LexA self-cleavage.

Differences in HI have been linked to a time-dependent induction of SOS genes, where genes with a low HI are expressed later in the SOS response (Goodman et al., 2016; Lima-Noronha et al., 2022). However, our data shows that despite significant differences in HI between cluster 1–3, the mean expression of these gene clusters are induced at 10 min (Figure 3A). This indicates that genes with an SOS box that binds LexA strongly will be derepressed simultaneously as genes with an SOS box that binds LexA weakly. Therefore, we propose that HI is mainly implicated in the level of gene expression after SOS activation and not the time of expression (Figure 3B).

### 3.5 The e14 prophage encoded genes are expressed late in the SOS response

While genes within cluster 1 to 3 are induced from 10 min, cluster 4 does not exhibit increased expression until 50 min (Figure 3A). The SOS inducible e14 prophage element genes found in cluster 4 are examples of phage DNA found in the bacterial genome which often have roles in stress tolerance and antibiotic resistance (Mehta et al., 2004; Wang et al., 2010). Such prophage elements often have separate regulatory systems, and e14 elements are regulated by a protein similar to the more known CI repressor in lambda phages (Mehta et al., 2004). Some CI repressors resemble LexA in that they bind the RecA-ssDNA nucleoprotein filament which induces self-cleavage. However, while RecA-ssDNA promotes a quick self-cleavage of LexA, the CI repressors undergo a slower self-cleavage (Butala et al., 2009). This delayed RecA-ssDNA mediated cleavage of CI repressors might explain why the e14 genes *intE, ymfJ* and *ymfM*, which are regulated by CI-like repressors, are expressed late in the SOS response (Figure 3A, Cluster 4). Additionally, the HI values of *intE* and *ymfJ* do not follow the correlation between HI and maximum LFC as seen for the LexA-repressed genes (Figure 3B). Even though these genes are listed as LexA-repressed genes (Simmons et al., 2008), the SOS box might not be as relevant for the expression of the e14 genes as they are under CI-like repression.

### 3.6 Genes encoding several functions of the SOS response are expressed simultaneously shortly after DNA damage

**Figure 4:**
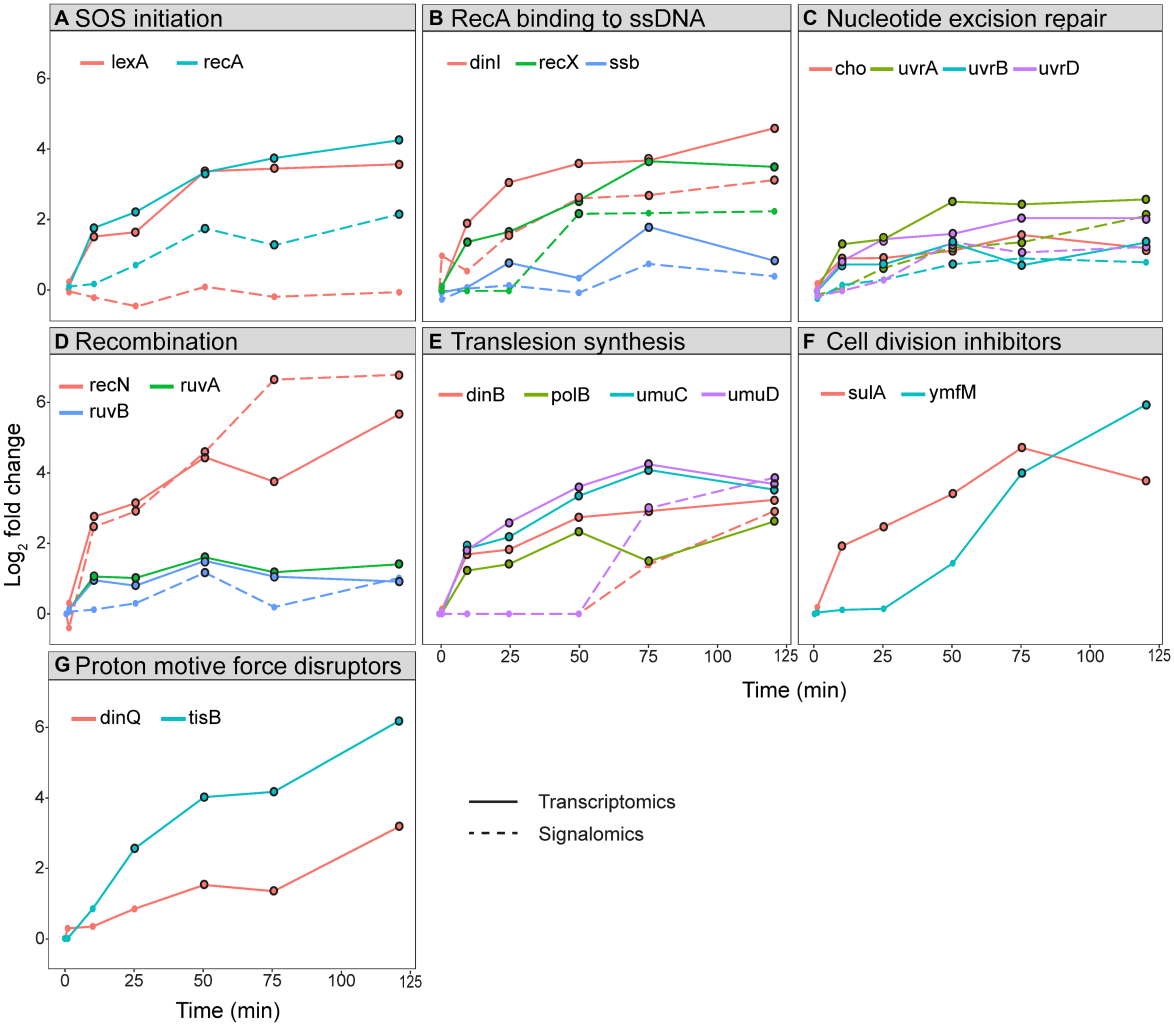
Log_2_ fold change of gene expression and protein activation in the SOS response. Log_2_ fold change of transcripts (straight line) and activated proteins (stippled line) during a 120 min sampling period after ciprofloxacin treatment in *E. coli*. The genes and proteins have been grouped based on function: **(A)** SOS initiation, **(B)** RecA binding to ssDNA, **(C)** nucleotide excision repair, **(D)** recombination, **(E)** translesion synthesis, **(F)** cell division inhibitors, and **(G)** proton motive force disruptors. Timepoints for genes with an FDR < 0.05 and proteins with an FDR < 0.1 have a black border.

The SOS response can be divided into functional groups to compare the longitudinal expression and protein activation within and between the different groups. The groups we have analyzed include SOS initiation, RecA binding to ssDNA, nucleotide excision repair, recombination, translesion synthesis, cell division inhibitors, and proton motive force disruptors (Figure 4).

The functional group called SOS initiation shows that expression of both *lexA* and *recA* are rapidly induced 10 min after ciprofloxacin treatment, but only activated RecA increased from 25 min (Figure 4A). As discussed above, levels of activated LexA remain unchanged due to self-cleavage. Most of the other genes and proteins in the SOS response follow the pattern of *recA*/RecA (Figures 4B–D).

The temporal activation of TLS polymerases is reported to start with PolB (Pol II) and DinB (Pol IV), followed by UmuD’_2_C (Pol V), and is coordinated by both transcriptional and post-translational regulation (Rangarajan et al., 1999; Robinson et al., 2015; Henrikus et al., 2018; Lima-Noronha et al., 2022). Our expression data did not confirm a delayed expression of *umuDC*, as all TLS polymerase genes were expressed at 10 min and had a similar expression profile (Figure 4E). This was verified in an independent batch cultivation study of ciprofloxacin-treated *E. coli* in mineral media (to be published elsewhere). Therefore, these results show that that the TLS polymerases are not temporally segregated at expression level. Increased pulldown of UmuD and DinB were detected first at 75 min (Figure 4E), i.e., post-translational regulation likely ensures their activation in the late phases of the SOS response and not directly after translation. This agrees with previous reports showing that DinB and UmuDC are regulated after translation to provide time for error-free repair prior to TLS (Godoy et al., 2007; Goodman et al., 2016). Notably, UmuD and DinB do not differentiate in time of activation in our results (Figure 4E). Despite detection of active UmuD, the MIB assay cannot determine whether this represents the functional Pol V; thus, the polymerase activity can still be delayed compared to Pol IV. Collectively, our results show that the TLS polymerases are not temporally segregated at expression level, and that any temporal differences in polymerase activity is likely due to post-translational regulation.

It has been argued that the SOS response relies on transcriptional regulation to ensure that error-free repair occurs before cell division inhibition and TLS (Janion, 2008; Simmons et al., 2008; Lima-Noronha et al., 2022). However, our transcriptomics data shows that nucleotide excision repair genes, the cell division inhibitor *sulA*, and TLS polymerases are all expressed at 10 min (Figures 4C,E,F). Therefore, temporal segregation of these processes at the expression level cannot be validated. Any longitudinal regulation appears to take place after transcription as activated proteins within error-free repair were detected directly after translation while activated proteins constituting TLS polymerases were only detected at later timepoints (Figures 4C,E). The inconsistency with previous studies might be due to our use of: (i) ciprofloxacin as the DNA damaging agent and at a dose that avoids extensive cell death, (ii) highly controlled growth conditions in bioreactors, and (iii) RNA sequencing, which is superior to microarray and gene reporter systems.

### 3.7 The membrane potential disruptors might cause decreased nucleotide metabolism, energy coupling, and motility

After the primary response of SOS activation, secondary effects appear from 50 min and include decreased expression of genes involved in nucleotide metabolism, energy coupling, and motility (Figure 1D). These effects can be regarded as general stress responses reflecting energy depletion of the cell in response to ciprofloxacin, similar to what has been reported for other antibiotics and general stressors (Shaw et al., 2003; Kaldalu et al., 2004; Cirz et al., 2006, 2007; Jozefczuk et al., 2010). Here, we assume that the observed secondary effects are a direct consequence of SOS activation. The LexA-repressed membrane potential disruptors TisB and DinQ, which are part of toxin-antitoxin systems, insert into the inner membrane and cause reduced ATP pools, membrane depolarization, and a slower growth rate (Unoson and Wagner, 2008; Weel-Sneve et al., 2013). This might reduce the cellular metabolism, leading to the observed decrease in the expression of genes regulating nucleotide metabolism, energy coupling, and motility (Figure 1D). The bacterial population could benefit from a reduced metabolism by forming a heterogenous population of dormant cells (i.e., persisters) which have increased tolerance to antibiotics. Indeed, both TisB and DinQ have been implicated in the formation of persister cells (Dörr et al., 2010; Weel-Sneve et al., 2013). Interestingly, the expression of *tisB* and *dinQ* continues to increase during the sampling period and reaches the highest LFC at 120 min (Figure 4G). As TisB and DinQ are small peptides that do not have a catalytic site which binds ATP/GTP, we could not detect them with the MIB assay.

### 3.8 DNA damage increased nucleotide pools to allow for efficient mutagenesis

**Figure 5:**
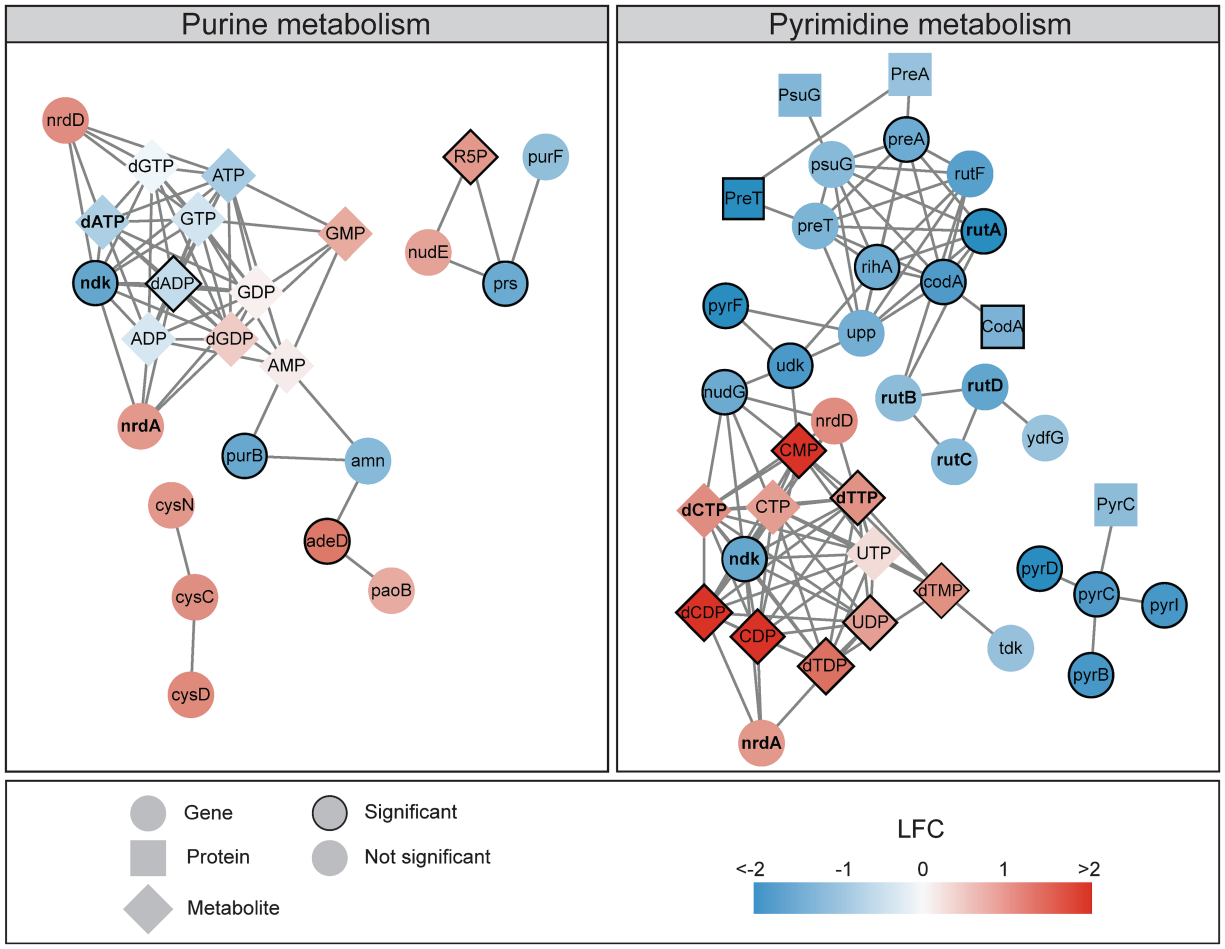
A multi-omics network of the nucleotide metabolism reveals decreasing gene expression and activated proteins and increasing metabolite pools 120 min after ciprofloxacin treatment. Networks illustrates the KEGG pathways of purine metabolism (eco00230) and pyrimidine metabolism (eco00240), colored according to the log2 fold change (LFC) at 120 min. Genes (FDR < 0.05 and LFC < −1 or >1), proteins (LFC < −1 or >1), and all metabolites are included in the networks. Significant genes (FDR < 0.05 and LFC < −1.5 or >1.5), proteins (FDR < 0.1), and metabolites (FDR < 0.05) have a border. Interactions within and between genes and metabolites are obtained from the KEGG pathways, and proteins are connected to their corresponding genes. Genes, proteins, and metabolites discussed in the text are bold.

Integrated transcriptomics, signalomics, and metabolomics revealed that DNA damage induced by ciprofloxacin affected nucleotide metabolism, causing decreased gene expression and increased pools of pyrimidine ribonucleotides and deoxyribonucleotides (NXP/dNXP, X = mono, di or tri; Figure 1D). Figure 5 highlights the major changes in purine and pyrimidine metabolism after ciprofloxacin treatment for 120 min. After temporary DNA damage induced by UV irradiation in *E. coli*, a reported increase in dNTPs was attributed to increased expression of the ribonucleotide reductases *nrdAB* that convert NXPs to dNXPs (Gon et al., 2011). In contrast to UV irradiation, ciprofloxacin causes persistent DNA damage throughout the treatment period as it is not broken down or metabolized by *E. coli*. Our study also showed increased *nrdA* expression after DNA damage, however, not significantly (Figure 5). Increased *nrdA* expression might contribute to the elevated pyrimidine pools detected here, but we propose two additional factors: (i) The gene *ndk*, which encodes Ndk and has the most metabolite interactions in the multi-omics network, has reduced expression at 120 min (Figure 5). Deletion of *ndk* in *E. coli* is reported to increase dCTP and decrease dATP pools (Lu et al., 1995; Schaaper and Mathews, 2013; Mathews, 2014), which is similar to our results (Figure 5). (ii) SOS activation slows down replication until the DNA damage is repaired and this could cause accumulation of nucleotides. However, while pyrimidine nucleotides are mainly used for incorporation into DNA and RNA, purine nucleotides are also used in signaling and energy metabolism. This could explain why only the pyrimidine nucleotide pools increased as elevated stress from DNA damage can yield a higher demand for purine nucleotides.

Treating yeast with a DNA-damaging agent increased dNTPs 6- to 8-fold, and it was suggested that elevated nucleotide pools led to more efficient TLS and thus higher survival. The highest increase in nucleotide pools after DNA damage in yeast was detected for dTTP and dCTP (Chabes et al., 2003), and this is in line with our results (Figure 5). In our study, concurrent downregulation of the pyrimidine breakdown genes *rutABCD* during the SOS response further supports a demand for increased nucleotide pools after DNA damage, possibly to yield higher survival (Figure 5). The largest impact on nucleotide pools was observed at 120 min, which coincides with more active TLS in the late phases of the SOS response (Figure 1C). It is also worth noting that increased dNTP pools in *E. coli* has been suggested to trigger mutagenesis independent of TLS by reducing the proofreading activity of Pol III (Gon et al., 2011).

## 4 Concluding remarks

In this study, we found that the expression of SOS genes for error-free and error-prone repair is induced simultaneously after ciprofloxacin treatment and that any temporal segregation occurs after transcription. Moreover, the HI is negatively correlated with maximum LFC, and not with the time of SOS gene transcription. These findings challenge previous studies that show a temporal regulation of SOS genes at the transcriptional level. The discrepancy from previous studies might be attributed to our use of ciprofloxacin as the DNA damaging agent and at a dose that avoids extensive cell death. Additionally, our results were produced by using highly controlled growth conditions in bioreactors and RNA sequencing, which is more sensitive compared to microarray and gene reporter systems. Finally, our data suggests that increased pyrimidine pools are part of a multilayered regulatory system, i.e., transcription, protein activation, and metabolite levels all facilitate efficient coordination of mutagenesis during the SOS response. This multilayered regulation of the SOS response is crucial for bacterial survival and is activated by several antibiotics. As SOS induced mutagenesis facilitates resistance development in bacteria, further research of the global SOS response at a clinically and ecologically relevant concentration might enable us to find new targets in the fight against resistance development.

## Data availability statement

The datasets presented in this study can be found in online repositories. The names of the repository/repositories and accession number(s) can be found in the article/Supplementary material.

## Author contributions

OB: Data curation, Formal analysis, Investigation, Methodology, Validation, Visualization, Writing – original draft, Writing – review & editing. AS: Data curation, Formal analysis, Investigation, Methodology, Validation, Visualization, Writing – original draft, Writing – review & editing. LR: Formal analysis, Investigation, Writing – review & editing. AB: Formal analysis, Software, Writing – review & editing. M-PS-B: Formal analysis, Software, Writing – review & editing. MR: Resources, Software, Writing – review & editing. AD: Supervision, Writing – review & editing. PB: Conceptualization, Data curation, Supervision, Writing – review & editing. MO: Conceptualization, Data curation, Funding acquisition, Project administration, Supervision, Writing – review & editing.

## Footnotes

^1^ https://www.uniprot.org/proteomes/UP000000625

^2^ https://www.ncbi.nlm.nih.gov/geo/query/acc.cgi?acc=GSE49682
