# Supplementary material for: SOS genes are rapidly induced while translesion synthesis polymerase activity is temporally regulated
Source: Front Microbiol. 2024 Mar 26;15:1373344. doi: 10.3389/fmicb.2024.1373344 (PMC11002266; doi:10.3389/fmicb.2024.1373344)
Supplement: Supplementary file 3 [file Table_3.DOCX]

----------

To review GEO accession GSE249682:

Go to <https://www.ncbi.nlm.nih.gov/geo/query/acc.cgi?acc=GSE249682>

Enter token kbodyewwvpgbfmn into the box

----------
