# Supplementary material for: SOS genes are rapidly induced while translesion synthesis polymerase activity is temporally regulated
Source: Front Microbiol. 2024 Mar 26;15:1373344. doi: 10.3389/fmicb.2024.1373344 (PMC11002266; doi:10.3389/fmicb.2024.1373344)
Supplement: Supplementary file 4 [file Data_Sheet_1.pdf]

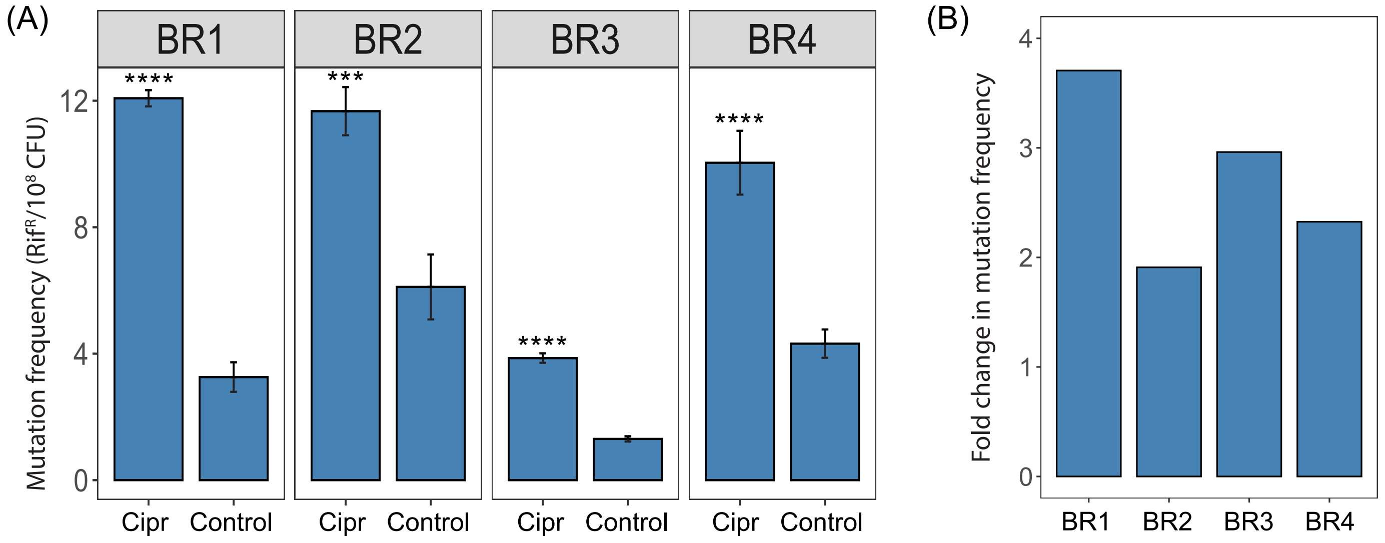

Supplementary Figure S1: Results from the rifampicin resistance (Rif^R) assay. Each biological replica (BR) consists of 3 technical replicas. (A) Mutation frequency (Rif^R/10^8 CFU) of ciprofloxacin (cipr, 12 ng/mL) treated *E. coli* and untreated control. Significance was calculated by using a two-tailed, Student's t-test (*** p < 0.001, **** p < 0.0001). Mean ± SD. (B) Fold change in mutation frequency of ciprofloxacin treated *E. coli* relative to the untreated control.

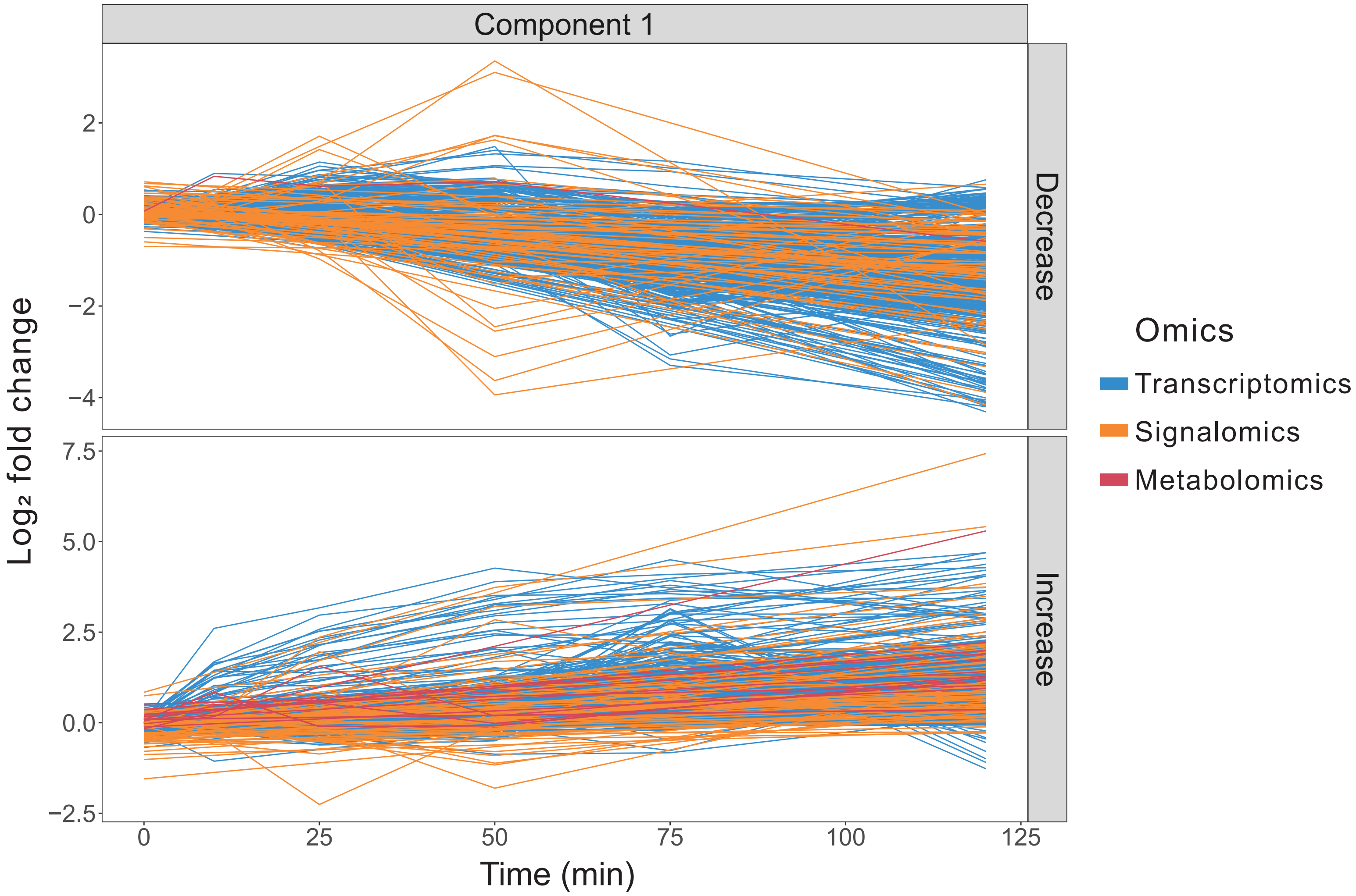

Supplementary Figure S2: Differentially expressed genes (1-50 min, FDR < 0.05; 75-120 min, FDR < 0.05, LFC < −1.5 or > 1.5), differentially enriched proteins (FDR < 0.1) and differentially enriched metabolites (FDR < 0.05) were modeled with linear mixed effect model spline and clustered with multi-block partial least squares. Two clusters within 1 principal component received the highest silhouette score of 0.50. The increasing cluster contained 615 transcripts, 79 activated proteins, and 13 metabolites while the decreasing cluster contained 478 genes, 89 activated proteins, and 1 metabolite.

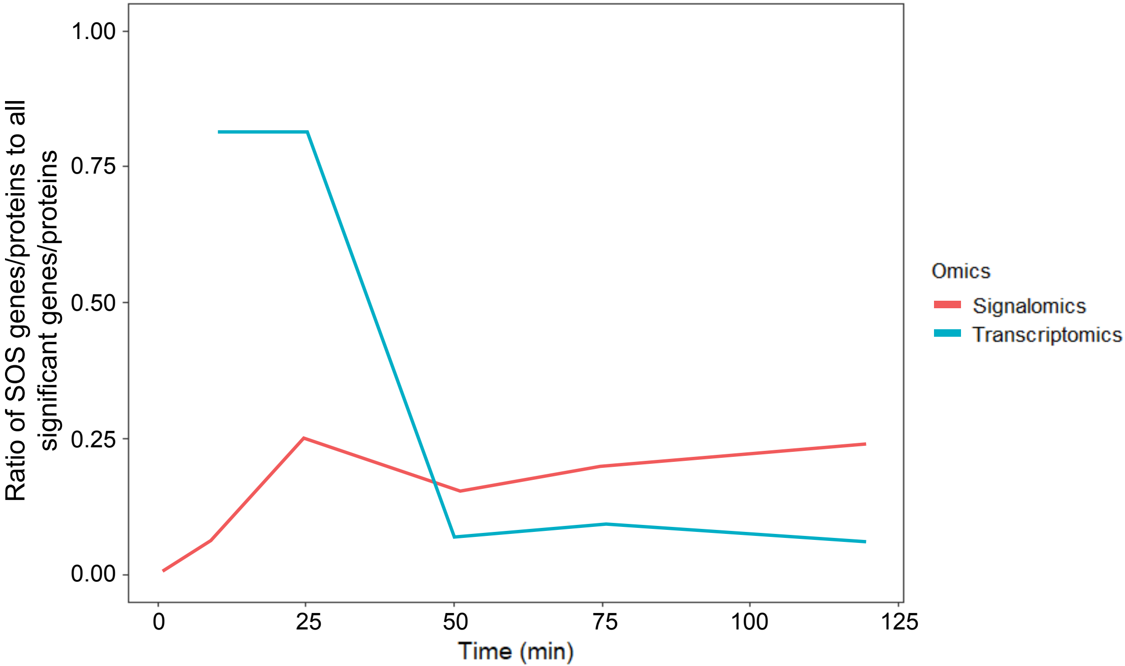

Supplementary Figure S3: The ratio of significant SOS genes or proteins relative to all significant genes or proteins at each sampling timepoint as detected by transcriptomics and signalomics.

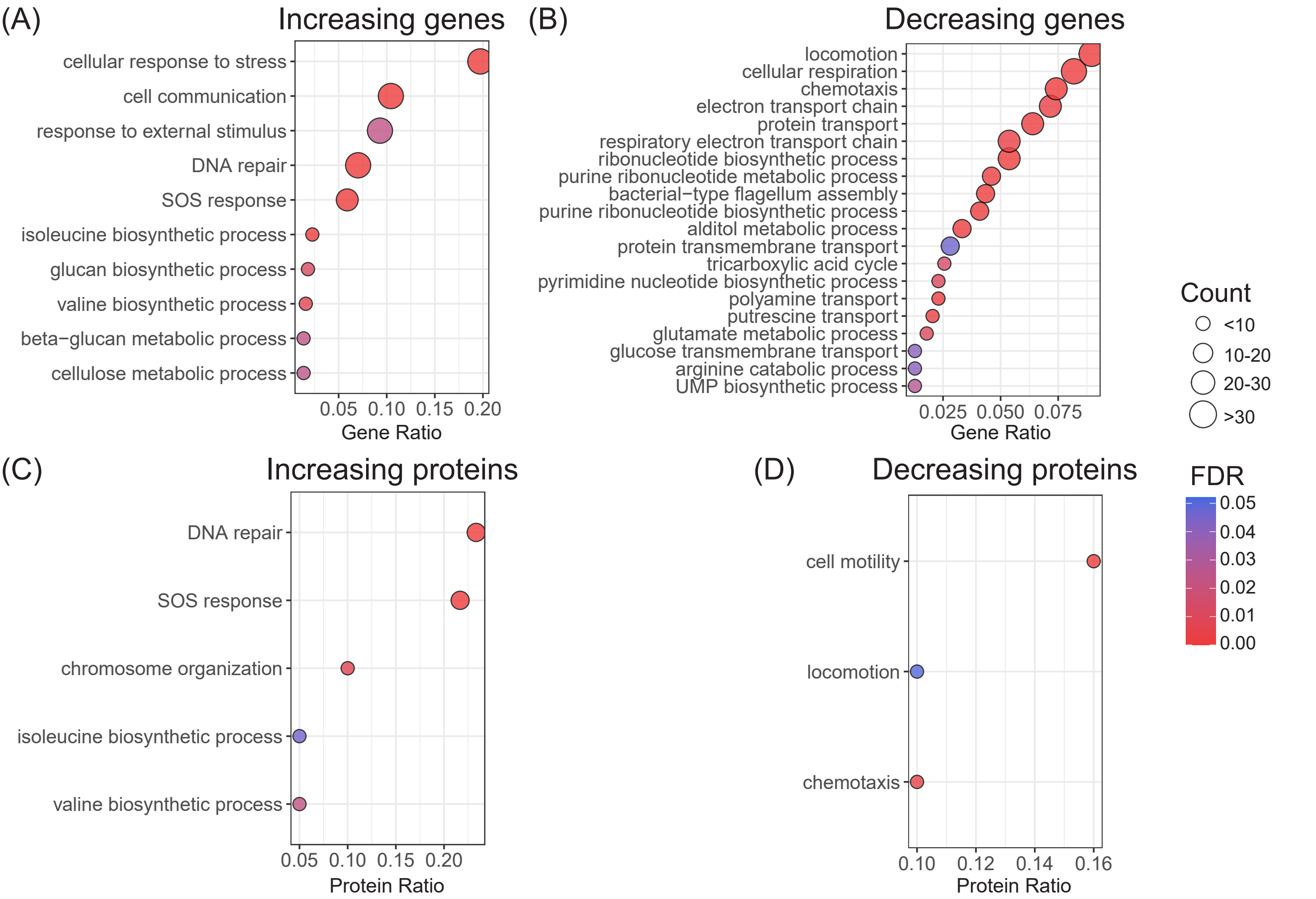

Supplementary Figure S4: A gene ontology (GO) analysis of genes and proteins from multi-block partial least squares clustering. The GO analysis was conducted using clusterProfiler from Bioconductor which uses Fisher's exact test to find overrepresented GO terms with FDR<0.05. Enriched GO terms for genes obtained from (A) the increasing cluster and (B) the decreasing cluster. Enriched GO terms for proteins obtained from (C) the increasing cluster and (D) the decreasing cluster.
